# Data mining of bulk and single-cell RNA sequencing introduces *OBI1-AS1* as an astrocyte marker with possible role in glioma recurrence and progression

**DOI:** 10.1186/s13148-022-01260-4

**Published:** 2022-03-08

**Authors:** Ali Mamivand, Shiva Bayat, Abolfazl Maghrouni, Sasan Shabani, Alireza Khoshnevisan, Hiva Saffar, Mina Tabrizi

**Affiliations:** 1 Department of Medical Genetics, School of Medicine, Tehran University of Medical Sciences, P.O. Box 14155‐6447, 14176‐13151 Tehran, Iran; 2 Department of Neurosurgery, Shariati Hospital, Tehran University of Medical Sciences, Tehran, Iran; 3 Department of Pathology, Shariati Hospital, Tehran University of Medical Sciences, Tehran, Iran

**Keywords:** RNF219-AS1, Astrocyte marker, Glial cell, Single-cell RNA sequencing (scRNAseq), Topologically associated domains (TADs)

## Abstract

Long non-coding RNAs (LncRNAs) are widely known for their various functions in cancer from tumor initiation to tumor progression and metastasis. Gliomas are the most prevalent primary forms of brain tumor, classified into grades I to IV according to their malignant histological features with grade IV, also known as glioblastoma multiforme (GBM), displaying the highest level of malignancy. Thus, the search for differentially expressed LncRNAs in GBM versus low-grade glioma to uncover new insights into the molecular mechanisms of glioma progression have intensified. Bulk RNA sequencing pinpointed decreased expression of *OBI1-AS1* in GBM compared to low-grade glioma samples. Subsequent single nuclei RNA sequencing revealed *OBI1-AS1* to be a super-exclusive astrocyte marker with AUC = 0.99 and the potential to fully differentiate astrocytes from other brain cell types. Additional supplementary bioinformatics analysis exhibited *OBI1-AS1* role in synaptic signal transduction and glutamatergic signaling. In addition, ChIP-Seq data were analyzed to explore transcription factors that can regulate *OBI1-AS1* expression in neural cells. Results of Hi-C, methylation and ChIP-Seq analysis strongly suggest methylation of the CTCF binding site serving a central role in regulation of *OBI1-AS1* expression via managing chromatin interactions. Our study indicated that lncRNAs, like *OBI1-AS1,* could be extremely precise in identifying the astrocyte cluster in the single-cell transcriptome and demonstrating superiority to well-established astrocyte markers such as *GFAP*, *S100B*, *ALDH1L1, and AQP4*.

**Graphical abstract:**

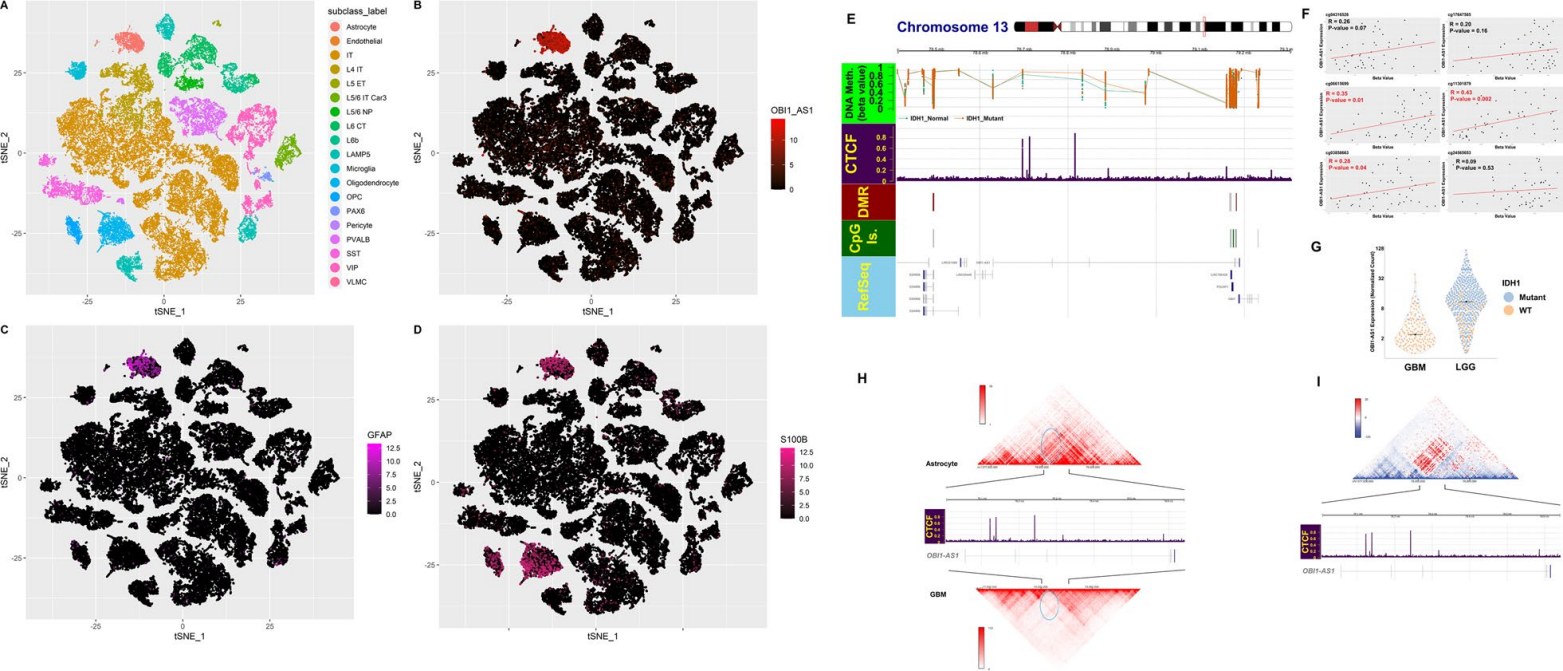

**Supplementary Information:**

The online version contains supplementary material available at 10.1186/s13148-022-01260-4.

## Introduction

Glioma is the most prevalent primary brain tumor. It is classified into grades I to IV by the World Health Organization (WHO) based on its malignant features with grades I, II and III classified as low-grade glioma (LGG) and grade IV classified as high-grade glioma (HGG) or known as glioblastoma multiforme (GBM) [1]. GBM has the highest mortality rate and the lowest 5-year survival rate in less than 10% of patients when compared with LGG being 26% in patients aged 55–64, 73% in patients aged 20–44 and 46% for those in the 45–54 age groups [2]. GBMs can either be primary (pGBM), arising de novo, or can occur as a consequence of LGG recurrence with higher grades, known as secondary GBM (sGBM). Primary and secondary GBM can differ in regards to their genetic and epigenetic landscape [3] with sGBM displaying more aggressive features and poorer prognosis than pGBM [4]. It is also noteworthy that progression of LGG to GBM increases the mortality rate [5], which makes this an important area of research to find key genes implicated in malignant progression of LGG to GBM for potential future therapeutic and interventional measures to enhance patient survival.

Recently, a massive group of genes coding for non-coding RNAs (ncRNAs) rather than proteins have been in the spotlight. Non-coding RNAs exert their functions either at the RNA level by inhibiting transcription of RNAs or at protein level by impeding translation [6]. These non-coding RNAs can be grouped based on the number of nucleotides they contain into small (Small non-coding RNA < 200 nucleotides) or long (Long non-coding RNA > 200 nucleotides). They play roles in tumorigenesis and tumor progression through DNA damage response, immune escape metabolic dysregulation of cancer cells that help provide the energy required for aberrant cell growth. Additionally, they are implicated in tumor metastasis by modulating epithelial-to-mesenchymal transition (EMT), a complex process through which cancer cells lose their epithelial features and gain mesenchymal features, enhancing their migration and invasive abilities [7]. *HOTAIR* is just one of a plethora of LncRNAs involved in glioma by promoting cell cycle progression and is tightly linked with poor prognosis [8]. In addition, recent studies suggest lncRNAs to be major players in glial cell microenvironmental dynamics. *LncRNA-ATB* is an example of an exosomal lncRNA that is implicated in astrocyte activation to finally lead to glioma cell invasion [9]. *LincRNA-GM4419* is yet another lncRNA implicated in promotion of trauma-induced apoptosis of astrocytes by upregulating TNFa [10]. These findings underscore lncRNAs to be among key regulatory elements in preserving brain homeostasis via glial cells.

In the present study, we conducted a systematic bioinformatics study utilizing The Cancer Genome Atlas (TCGA) database for analysis of differentially expressed LncRNAs (DELncRNAs) in LGG and GBM samples in hopes of finding potential LncRNAs that might play a role in transformation of LGG to GBM. This analysis showed that *OBI1-AS1* is one of the most significant DELncRNAs downregulated in GBM. Real-time PCR confirmed this finding experimentally. Using the GEPIA web server, we also investigated the existing correlation between *OBI1-AS1* expression and overall survival of patients. Gene Ontology and pathway enrichment analyses were performed to underpin molecular functions, biological processes, cellular components and signaling pathways involving *OBI1-AS1*. In addition, we analyzed single-cell RNA-Seq data to find cell types expressing *OBI1-AS1*. Our results revealed this gene to be only expressed in astrocytes. Astrocytes, the most abundant glial cell type in the central nervous system, fulfill numerous essential functions contributing to formation of the blood–brain barrier, synaptogenesis, maintenance of ion homeostasis, neurotransmitter buffering, and secretion of neuroactive agents and intracellular calcium signaling [11]. Also, single-cell RNA sequencing (scRNAseq) confirms the decreased expression of *OBI1-AS1* in GBM samples. In addition, assessment of methylation level in neighboring region of the gene displayed extreme hypermethylation in LGG. Finally, ChIP-Seq analysis was performed to elucidate transcription factors in close contact with the *OBI1-AS1* promoter to clarify interacting proteins contributing to regulation of *OBI1-AS1* expression and, therefore, playing their part in glioma malignant progression. Our findings suggested that *OBI1-AS1* has a potential role in synaptic signal transduction and we propose that *OBI1-AS1* may be potentially regulated by CTCF via restructuring topologically associated domains (TADs).

## Materials and methods

### Data availability

The sample datasets downloaded and processes for the current study included TCGA-LGG and TCGA-GBM for RNA-seq data [12], Allen Institute for Brain Science for scRNAseq (Normal brain) data [13, 14], ENCODE project for Hi-C and ChIP-Seq [15], CPTAC-3 for scRNAseq (GBM) data and TCGA-LGG and TCGA-GBM for Illumina HumanMethylation450 (450 k) data [12]. Sample IDs from each dataset were provided in the Excel file in Additional file 1.

### Bulk RNA sequencing analysis

GBM and LGG RNA-seq datasets and their associated clinical information were obtained from the TCGA database [12]. 40 GBM and 80 LGG samples were randomly selected for Differential Expression Analysis (DEA). Transcriptome data were analyzed using the edgeR package [16]. The *p* values were adjusted using the Benjamini–Hochberg method [17]. Subsequently, differentially expressed LncRNAs were chosen for further investigations and a volcano plot was created to visualize the DELncRNAs utilizing the R Enhanced Volcano package [18] based on False Discovery Rate (FDR) and Log 2 Fold Change (Log2FC).

### Survival analysis

**Fig. 1:**
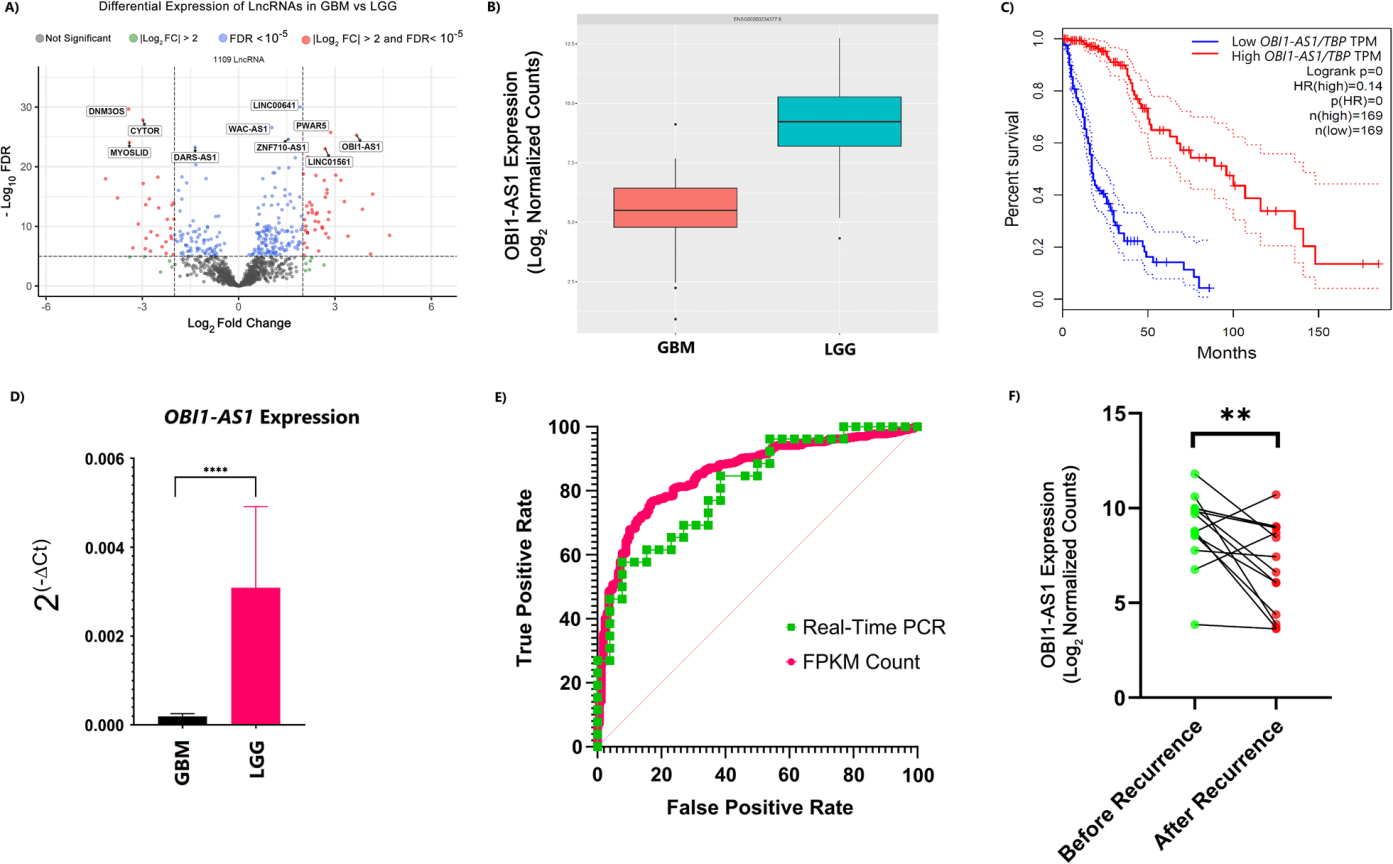
**A** In this volcano plot, differentially expressed LncRNAs in GBM compared to LGG are presented. The names of the top 10 with the least FDR are displayed in the figure with *OBI1-AS1* having the highest |Log2FC|. Positive values on the x-axis indicate increased expression in LGG vs GBM. **B** RNA-Seq Analysis of 120 glioma samples revealed decreased of *OBI1-AS1* expression in GBM compared to LGG by Log2FC = − 3.67. **C** Based on survival analysis of 338 glioma patients, those with low expression of *OBI1-AS1* exhibited significantly poorer prognosis with Hazard Ratio (HR) = 0.14. **D** Real-time PCR results confirmed our DEA findings. As is presented*, OBI1-AS1* was overexpressed in non-GBM glioma samples by log2FC = − 3.54 which was in accordance with our RNA-Seq data analysis findings (*p* value < 0.0001). **E** The ROC curve illustrated AUC equal to 81% and 85% for real-time PCR and RNA-seq data, respectively, with 52 samples (26 for GBM and 26 for LGG) for real-time PCR and 698 for RNA-seq investigation. These AUCs also highlight agreement between RNA-seq and real-time PCR findings. **F** Expression of *OBI1-AS1* decreased after recurrence (*p* value = 0.005). In 12 of the 14 patient, *OBI1-AS1* expression decreased after recurrence

GEPIA web server was used for analysis of any correlation between *OBI1-AS1* gene expression level and patient overall survival [19]. Patients were grouped into high expression and low expression categories according to whether their *OBI1-AS1* gene expression level was above the first quartile or below the third quartile, respectively. Consequently, Kaplan–Meier survival analysis was performed for the survival data as presented in Fig. 1C.

### ChIP-seq data analysis

The ChIP-Atlas-Enrichment Analysis online tool [20] was used to find the transcription factors (TF) binding to − 3000 < TSS < 3000 of *OBI1-AS1* transcription start site (TSS) in neural cells. The significance threshold was selected as greater than 100 based on peak caller MACS2 score (− 10Log10 [MACS2 Q-value]) which means that peaks with MACS2 Q-value (FDR) lower than ${10}^{-10}$ were considered. Moreover, CTCF and histone modifications’ bigwig file was downloaded from the National Bioscience Database Center (NBDC) and the ENCODE project [15, 21].

### Single nuclei RNA sequencing

We downloaded gene expression matrix, 2D coordinates, and trimmed median of gene expression in each cluster of brain cells from the Allen Institute for Brain Science [13, 14]. The whole QC criteria, cell type clustering and cluster annotation procedure were explained in detail by Hodge et al. [22]. In brief, raw data were aligned with GRCh38 and GRCh38.p2 as the reference genome and RefSeq transcriptome, respectively. Count table was created by applying summarizeOverlaps function to BAM files. Subsequently, cells with any one of the following criteria were filtered out from downstream analyses: less than 500,000 reads mapped to exonic or intronic sequences; less than 40% of total reads aligned to the reference genome; less than 30% cDNA longer than 400 base pairs; TA nucleotide ratio below 0.7 and less than 50% of unique reads. After removal of poor-quality cells based on mentioned parameters, genes located on sex and mitochondrial chromosomes were excluded to avoid false clustering based on sex and nuclei quality in subsequent analyses. Next, clusters were built and identified by the Louvain algorithm using top 20 principal components. Subsequently, clusters were annotated manually using panel of markers. To evaluate the upstream analysis, trimmed median of gene expression was used to create dendrogram of cell types by hierarchical clustering. Subsequently, t-SNE was drawn by 2D coordinates. We also used expression of *GFAP*, *S100B*, *ALDOC*, *SLC1A3*, *AQP4*, and *ALDH1L1* as well-known astrocyte markers to confirm astrocyte cluster identity [23, 24]. To normalize the gene expression matrix, CPM was applied to the read counts. To evaluate specificity of *OBI1-AS1* as an astrocyte marker, ROC curve analysis was performed based on CPM expression for *OBI1-AS1*, *GFAP*, *S100B*, *ALDOC*, *SLC1A3*, *AQP4*, and *ALDH1L1* across all cells.

#### Single-cell RNA sequencing (GBM)

Seurat object of GBM samples from the CPTAC-3 project was used for downstream analysis [25, 26]. IDs of samples used in this study are presented in Additional file 1. Initially, low quality cells were filtered out by calling isOutlier function from scater package in R [27]. Afterward, single-cell object was built in Seurat v4, and t-SNE was computed by top 30 principal components [28]. Next, clusters were generated by the Louvain algorithm using the nearest 20 neighbors. Top markers for each cluster were obtained using FindAllMarkers function from Seurat v4. Clusters were manually annotated by cell type based on the expression pattern of the markers.

### Patient samples for qRT-PCR with IHC diagnosis

26 GBM and 26 LGG samples were collected from Shariati Hospital affiliated with Tehran University of Medical Sciences (TUMS). All samples were primary tumors without any reported recurrence. There were 17 and 14 males in GBM and LGG groups, respectively. Pathological diagnosis and immunohistochemical analysis of the tumor type was carried out by an expert neuropathologist based on the World Health Organization classification of tumors (grade I to IV). The histopathological diagnosis of the obtained tissue samples was conducted based on immunohistochemical detection of the following proteins: GFAP, OLIG2, p53, KI67, IDH1, ATRX, and EGFR. All samples were evaluated by immunohistochemical and real-time PCR analyses. Tumor samples were all collected in RNA Later immediately after surgical resection and stored at − 80° centigrade until RNA extraction. Written informed consent was obtained from all patients enrolled in this study. This study fully conformed with ethical standards of Tehran University of Medical Sciences and the 1975 Helsinki Declaration.

### RNA extraction, cDNA synthesis and quantitative RT-PCR

RiboEx™ (GeneAll) was used for RNA extraction based on manufacturer’s protocol. Presence of genomic contamination was checked by agarose gel electrophoresis before cDNA synthesis. RNA concentration and presence of contaminants were determined using the NanoDrop 2000 spectrophotometer (Thermo Scientific). cDNA synthesis was performed using the PrimeScript RT reagent (TakaraBio Inc, Shiga, Japan) and qRT-PCR was carried out using the AMPLIQON Real Q Plus 2 × Master Mix Green low ROX in the Light Cycler® 96 System (Roche Life Science, Germany) based on the manufacturer’s instructions. Real-time PCR was conducted in duplicates. Primers were designed using the Oligo software and were blasted to check their specificity afterward. Primer sequences are presented in Table 1. Standard curves were created to set-up the primers and the primers were set-up with efficiency equal to 2. Relative quantification of target gene expression was performed using the 2^–∆∆**Ct**^ method with *B2M* as the normalizer gene. The real-time procedure for each sample was as follows:

**Table 1:** Primer sequence

Gene	Forward primer	Reverse primer
*OBI1-AS1*	GCCCTGAAGCATACCAAAATGT	CACAGAAAGTACCCAAGAGGT
*B2M*	AGATGAGTATGCCTGCCGTG	GCGGCATCTTCAAACCTCCA

Incubation for 10 min at 95 °C followed by 40 cycles of elongation including 10 s at 95 °C and 30 s at 60 °C. To exclude presence of any primer dimers or by-products, dissociation curves were carefully analyzed to check the specificity of the product melting peak. The PCR products were ultimately confirmed by 2% agarose gel electrophoresis.

### Statistical analysis

The Q-Q plot was used to assess normal distribution of the data. The Mann–Whitney test was carried out for comparison of groups. The ROC curve and the area under the curve (AUC) were used to evaluate the sensitivity and specificity of the LncRNA in distinguishing GBM from LGG. A *p* value less than 0.05 was deemed statistically significant for a confidence interval of 95%. Statistical analysis was performed with GraphPad Prism8.

### Methylation

Illumina HumanMethylation450 (450 k) array data, which are available in Genomic Data Commons (GDC), have been used in the current study. Initially, matrix of beta-values for the samples was downloaded, and probes belonging to the sex chromosomes or those known to have common SNPs at CpG sites were removed. Additionally, SNP probes with minor allele frequencies greater than 0.05 besides those mapping to multiple locations in the genome (cross-reactive probes) were filtered out [29]. After probe filtering, M-values for further statistical analyses were calculated. To find differentially methylated CpGs (DMCs) between LGG and GBM, linear model on matrix of M-values in limma was applied [30]. The M-values matrix was then annotated and analyzed by DMRcate to find differentially methylated regions (DMRs) [31]. DMRs represent multiple proximal CpG sites in the genome that are differentially methylated between groups. Eventually, all *p* values were adjusted by the Benjamini–Hochberg method and the results with FDR < 0.001 were considered significant.

### Gene ontology (GO) and pathway enrichment

We used *OBI1-AS1* co-expressed genes to perform functional annotation. A list of *OBI1-AS1* co-expressed genes from lncHub (https://maayanlab.cloud/lnchub/) was obtained [32]. lncHub computes mRNA-lncRNA correlations using the read counts of 11,284 TCGA RNA-seq samples processed by Recount2. Read counts were normalized by quantile, and, subsequently, Pearson’s correlation coefficient was computed across all genes. We selected genes with a Pearson correlation coefficient > 0.4 for further functional analysis. Enrichment analysis for biological process, molecular function, cellular component and pathways involved were conducted by DAVID [33, 34].

## Results

### Bulk RNA-seq data indicate that expression of OBI1-AS1 in GBM is lower than LGG

In this study, 728 LncRNAs exhibited differential expression between LGG and GBM (FDR < 0.05). These LncRNAs were further filtered for |Log2 Fold change|> 2 and this reduced the number of validated DELncRNAs to only 84 (Additional file 2). Subsequently, the top 10 DELncRNAs with the lowest FDRs were prioritized and selected for further investigations as presented in the volcano plot in Fig. 1A. *OBI1-AS1* was chosen because it displayed the highest level of |Log2 Fold change|= 3.67 (Fig. 1B). Furthermore, high expression level of this gene showed tight association with prolonged patient survival period as illustrated in Fig. 1C. Interestingly, thus far, no previous studies or investigations were conducted on this gene. To further validate the results obtained from our bioinformatics analyses, 26 GBM and 26 LGG tumor samples were collected and checked utilizing the qRT-PCR technique for confirmation of RNA-Seq findings. qRT-PCR confirmed lower expression of *OBI1-AS1* in GBM samples compared with LGG. The relative expression level of this gene is illustrated in Fig. 1D. Real-time PCR showed that Log2FC was − 3.54 which was completely compatible with our DEA results (Log2FC = − 3.67). To analyze the sensitivity and specificity of *OBI1-AS1* in distinguishing GBM from LGG, ROC curve was created and area under the curve (AUC) was measured (Fig. 1E). In addition, we performed differential expression analysis using all recurrent LGG samples (14 samples) and their pair before recurrence (before and after recurrence). The results showed that *OBI1-AS1* was downregulated after recurrence by FDR = 0.005 and Log2FC = − 2.11(Fig. 1F, Additional file 3).

### Single nuclei RNA sequencing (snRNA-Seq) analysis revealed that OBI1-AS1 is a super-exclusive marker for astrocytes

**Fig. 2:**
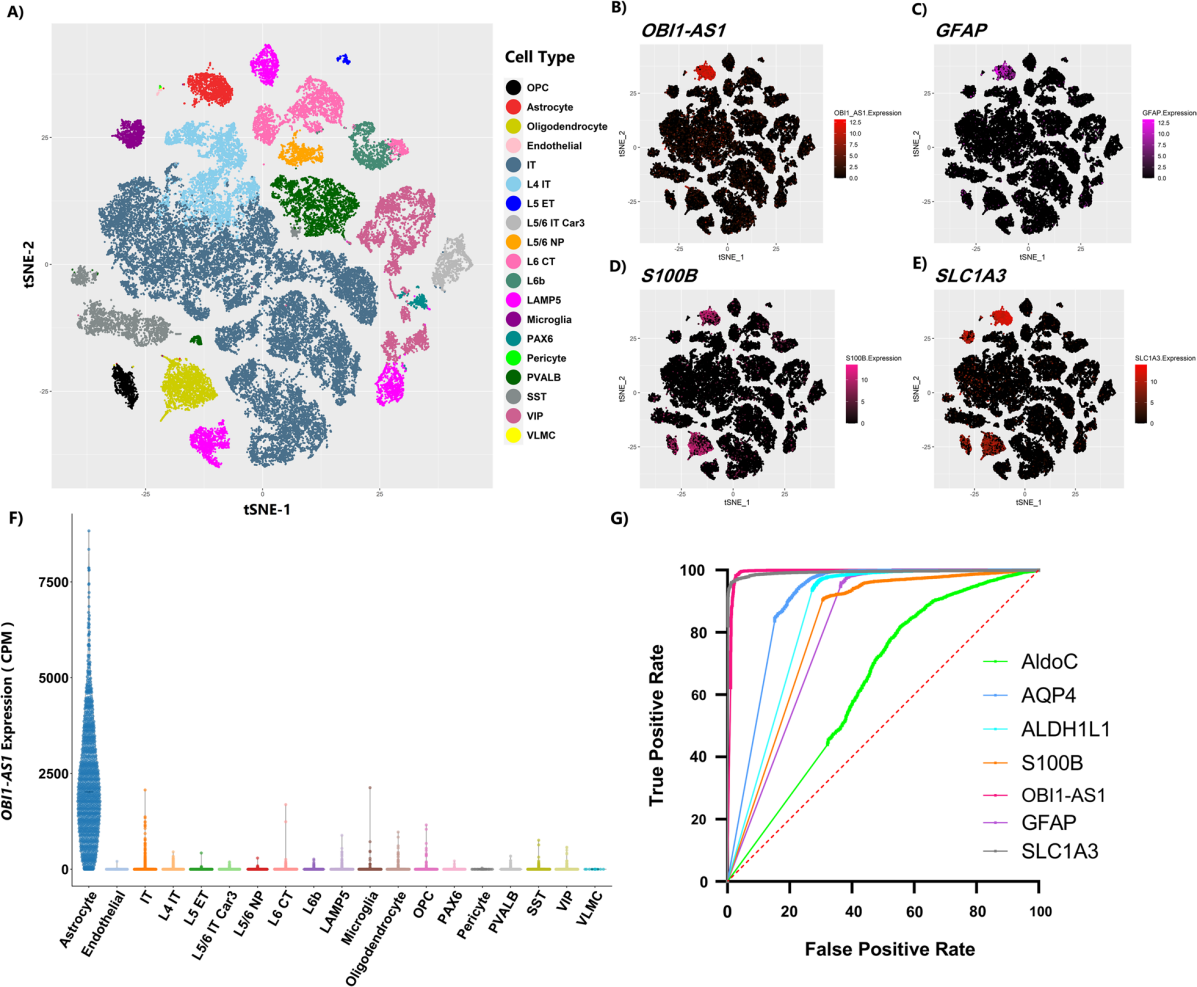
**A** t-SNE of 19 brain cell types. Each point is a cell. Red cluster belongs to astrocytes. **B**–**E** The expression pattern of astrocyte markers including *GFAP* (**C**), *S100B* (**D**) and *SLC1A3* (**E**) confirmed that the red cluster in **A** belonged to the astrocyte. *OBI1-AS1* expression was also plotted in t-SNE (**B**), and the pure red color in this figure indicated that *OBI1-AS1* is exclusively expressed in astrocytes. **F** Violin graph shows that *OBI1-AS1* is significantly expressed in astrocytes. Each point is a cell. **G** ROC curve analysis of *GFAP, S100B, SLC1A3, ALDOC, AQP4, ALDH1L1,* and *OBI1-AS1* showed that *OBI1-AS1* had significantly higher specificity and sensitivity compared to other markers for differentiating astrocyte from other cell types

Different cell populations in snRNA-Seq of normal brain are illustrated with t-SNE in Fig. 2A. As presented in Fig. 2B and Additional file 10: Figure S1, *OBI1-AS1* is exclusively expressed in astrocytes. Co-expression of *GFAP, S100B* and *SLC1A3*, well-established astrocyte markers in the red cluster, confirms that this cluster belongs to astrocytes (Fig. 2C–E). Other markers were presented in Additional file 11: Figure S2. The expression pattern of *OBI1-AS1* across all cell types is plotted in Fig. 2F. In Fig. 2G, ROC curve analysis was presented to assess *OBI1-AS1* and six other outstanding astrocyte markers’ power to differentiate astrocytes from other brain cells. As illustrated, *OBI1-AS1* was significantly more accurate than the other markers. To further clarify this point, comparison between Fig. 2B, D demonstrated that *S100B* has high expression in both oligodendrocytes and astrocytes, while *OBI1-AS1* was expressed exclusively in astrocytes. The area under the curve (AUC) and *p* values for these genes are shown in Table 2. Remarkably, AUC for *OBI1-AS1* was equal to 0.99, introducing this gene as a very specific marker for astrocytes.

**Table 2:** AUCs of Astrocyte markers in single-cell RNA-Seq

Marker name	AUC	*p* value
*OBI1-AS1*	0.99	< 0.0001
*SLC1A3*	0.99	< 0.0001
*GFAP*	0.81	< 0.0001
*ALDH1L1*	0.85	< 0.0001
*AQP4*	0.90	< 0.0001
*S100B*	0.81	< 0.0001
*ALDOC*	0.63	< 0.0001

### Single-cell RNA sequencing of GBM samples showed low expression of OBI1-AS1 in GBM cells

**Fig. 3:**
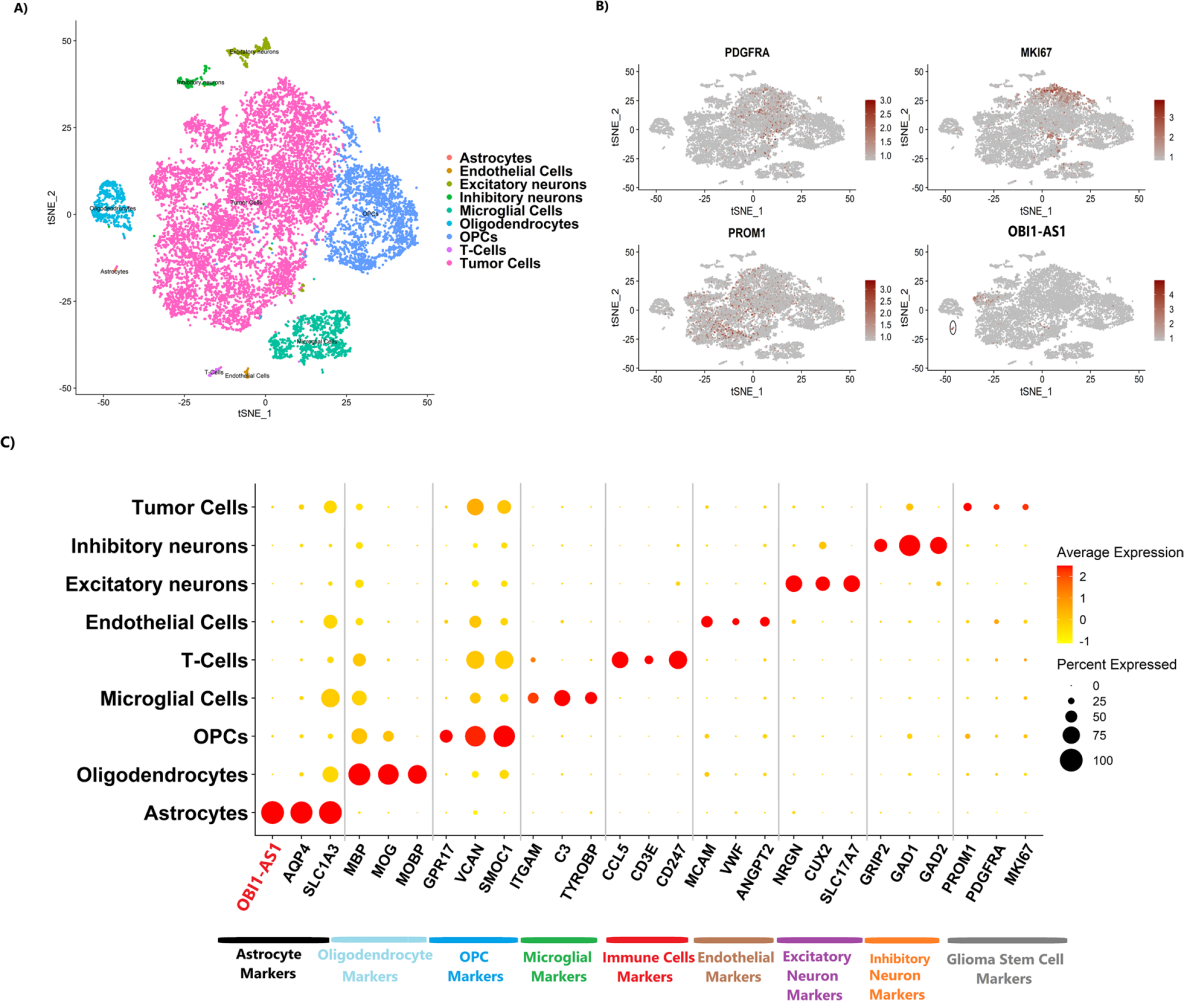
**A** t-SNE plot of GBM sample. **B** Feature plot for glioma stem cell markers(*PROM1, PDGFRA, MKI67*) and *OBI1-AS1*. Note that stemness markers are scattered throughout the tumor cluster. **C** Cells in **A** were manually annotated by cell type based on marker expression. As shown, *OBI1-AS1* was expressed only in the astrocyte cluster and not in tumor cells

Subsequently, GBM scRNAseq data were further analyzed to ascertain cell types in GBM tumors demonstrating elevated expression of *OBI1-AS1*. Our study showed that expression of *OBI1-AS1* in tumor cells was very low. In most patients, expression of this gene was not observed in any of the clusters (Additional file 12: Figure S3). In one patient (Fig. 3A), this gene showed high expression only in one small cluster (Fig. 3B, C). As shown in Fig. 3C, this small cluster mainly expresses astrocytic markers *(AQP4, SLC1A3)*. A complete list of cluster markers for this dataset is available in Additional file 4. Evaluation of markers for the *OBI1-AS1* expressing cluster in different databases shows that most of these genes had the highest expression in astrocytes. Actually, astrocyte markers separate this cluster from other cell populations. Since these cells are present in small distinct clusters, they seem unlikely to be a derivative of tumor cells; hence, they seem to be normal astrocytes in the tumor microenvironment. This finding was consistent with qRT-PCR and RNA-Seq results because our previous results showed very low expression of this gene in GBM tumors.

### OBI1-AS1 locus was hypermethylated in low-grade gliomas

Certainly, DNA methylation plays a pivotal role in regulation of gene expression, which encourages us to evaluate methylation to discover any possible association between *OBI1-AS1* expression and methylation. Surprisingly, this locus was found to be markedly hypermethylated in LGG. Our analyses revealed three differentially methylated regions (DMRs) with significant hypermethylation in LGG. The most significant was located upstream of *OBI1-AS1*, displaying overlap with a CpG island. This was an unexpected finding because it was in stark contrast with *OBI1-AS1* upregulation in LGG while hypermethylation usually suppresses gene expression. Statistical details for each DMR are available in Table 3. A complete list of DMRs and differentially methylated CpGs (DMCs) is available in Additional file 5.

**Table 3:** Statistical details for DMRs between LGG and GBM

Chromosome	Start	End	Width	Strand	no.CpGs^a^	min_smoothed_fdr^b^	Stouffer^c^	HMFDR^d^	Fisher^e^	Maxdiff^f^	Meandiff^g^	Overlapping.genes
chr13	78491982	78494462	2481	*	49	4.97E−154	2.40E−122	2.52E−10	3.81E−137	0.335893	0.086379	OBI1-AS1, EDNRB
chr13	79168044	79168573	530	*	6	3.38E−45	8.34E−37	1.81E−09	4.87E−35	0.343167	0.220062	OBI1-AS1
chr13	79181440	79184529	3090	*	14	1.88E−28	4.46E−21	4.91E−08	9.10E−31	0.294686	0.02905	OBI1-AS1

^a^no.CpGs: Number of constituent CpG sites of DMR

^b^min_smoothed_fdr: Minimum FDR of the smoothed estimate

^c^Stouffer: Stouffer summary transform of the individual CpG FDRs

^d^HMFDR: Harmonic mean of the individual CpG FDRs

^e^Fisher: Fisher combined probability transform of the individual CpG FDRs

^f^maxdiff: Maximum differential/coefficient within the DMR

^g^meandiff: Mean differential/coefficient across the DMR

### Hypermethylated CpGs demonstrated significant overlap with the CTCF binding site in midpoint of OBI1-AS1

**Fig. 4:**
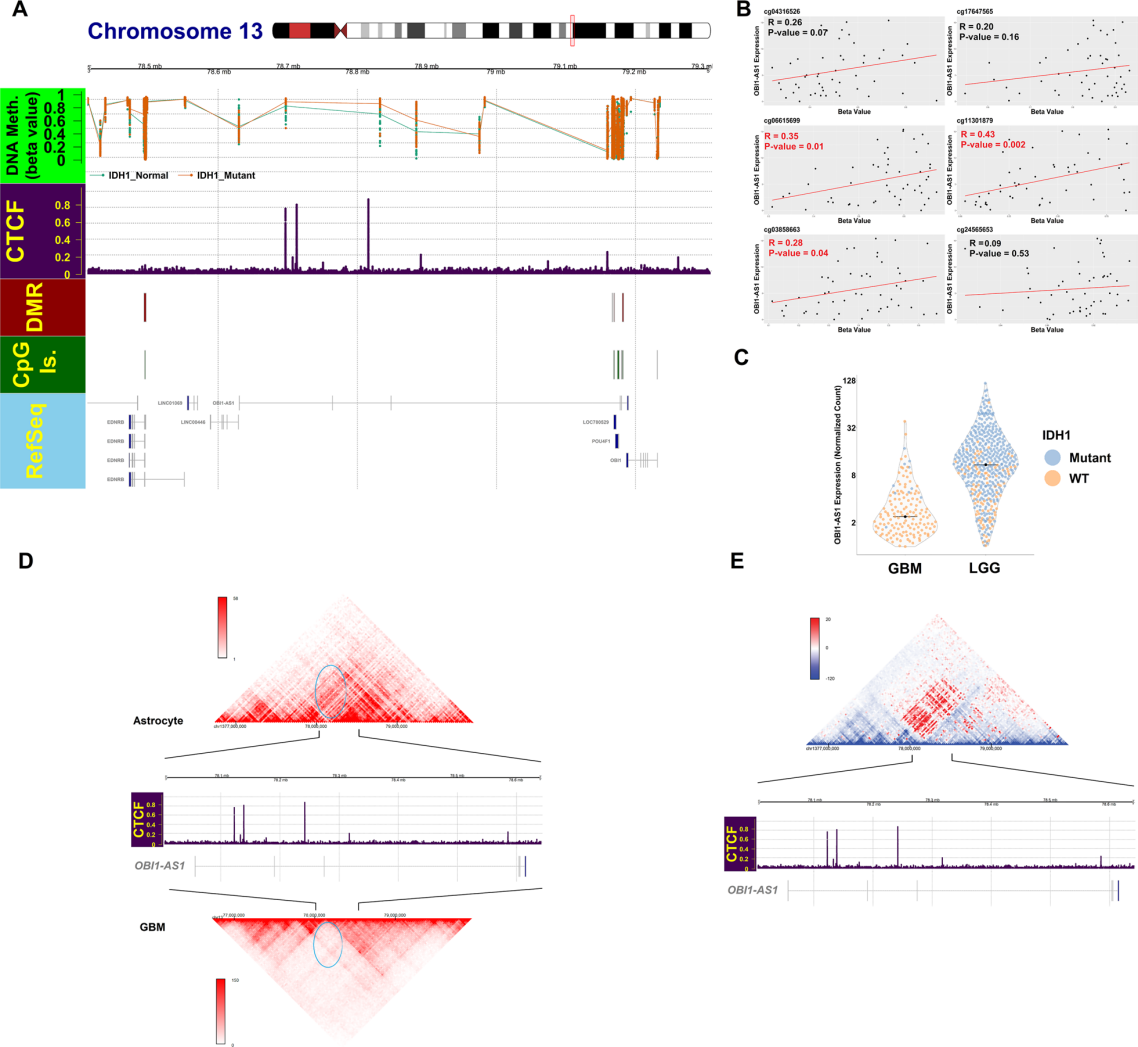
**A** Methylation status of probes between IDH1^wt^ LGG and IDH1^Mut^ LGG at *OBI1-AS1* locus. Most probes were hypermethylated in IDH1^Mut^ samples (orange point and line). Probes located in the intronic region of *OBI1-AS1* showed significant overlap with CTCF binding sites based on glioma ChIP-Seq data. **B** Correlation of *OBI1-AS1* expression with probe methylation. As is presented, there was a positive correlation between *OBI1-AS1* expression and methylation of probes that are in overlap with the CTCF binding site. **C** *OBI1-AS1* expression in GBM and LGG based on IDH1 mutation status. Cells were colored in orange and blue for IDH1^wt^ and IDH1^mut^, respectively. Median of each group was shown by a black line. Expression was higher than median in IDH1^mut^ samples in each group. **D** Hi-C interactions are shown for a 3-Mb region comprising *OBI1-AS1*. In GBM, a strong TAD boundary in the intronic region of *OBI1-AS1* (blue circle) was seen while this boundary site was eliminated in *OBI1-AS1*-expressing cells (astrocytes). There are multiple binding sites for CTCF at the boundary region. **E** Hi-C interactions for astrocytes and GBM are shown in subtracted heatmap. Hot color indicates areas in close contact in astrocytes compared to GBM. The reddest region was located precisely in the middle of *OBI1-AS1*, where in GBM, CTCF binds DNA with a high affinity. This finding confirmed the close contact between *OBI1-AS1* promoter with its downstream region in astrocytes, while CTCF prevented this contact in GBM

The question raised at this point was whether there was the possibility of *OBI1-AS1* being expressed at higher levels as a consequence of DNA hypermethylation in the region. Recent studies revealed that *IDH1* mutant gliomas showed CpG island methylator phenotype (CIMP) [35]. Given this, it is possible that IDH1 mutations may also affect the methylation of this region and expression of *OBI1-AS1*. To evaluate this, LGG samples were divided into normal and mutant groups based on *IDH1* mutation status and DEA and methylation analysis was performed. As expected, CpGs were hypermethylated in *IDH1*^mut^ samples at this locus (Fig. 4A). Also, DEA revealed *IDH1*^*mut*^ samples displayed higher levels of *OBI1-AS1* expression (Log2FC = 1.4 and FDR = ${2.9*10}^{-15}$) (Fig. 4C and Additional file 6). In addition, Methylation of the three probes demonstrated a significant positive correlation with *OBI1-AS1* expression (Fig. 4B). These findings suggest that these CpGs may play a role in regulating *OBI1-AS1* expression. ChIP-seq analysis of glioma samples showed that CTCF has several strong binding sites surrounding *OBI1-AS1* (Fig. 4A and Additional file 7). Interestingly, these sites overlap with DMCs that are significantly hypermethylated in *IDH1*^mut^ LGG samples (Fig. 4A and Additional file 8).

CTCF is considered as a well-known insulator with a crucial role in formation of topological associated domains (TADs). Therefore, organization of TADs in cells expressing *OBI-AS1* and also in GBM cells was analyzed to see whether CTCF is implicated in regulation of chromatin interactions at this locus. To accomplish this, Hi-C data of GBM and astrocytes publicly available in the 3D genome browser were used [15, 36, 37]. A comparison is presented in Fig. 4D, E. A strong TAD boundary at midpoint of *OBI1-AS1* in GBM Hi-C was seen. This is where the CTCF binding site is located and divides *OBI1-AS1* into two different TADs. As demonstrated, this boundary prevents interaction of upstream areas with the downstream sequence in GBM. In astrocytes, on the other hand, a lot of interaction at the same region was seen. This is more obvious in Fig. 4E. Hot colors in this figure indicate more interactions in astrocytes compared to GBM. Additional file 13: Figure S4 shows TADs along with histone modifications, CTCF ChIP-seq and chromatin accessibility at this locus.

### Potential role of OBI1-AS1 in synaptic signal transduction

**Fig. 5:**
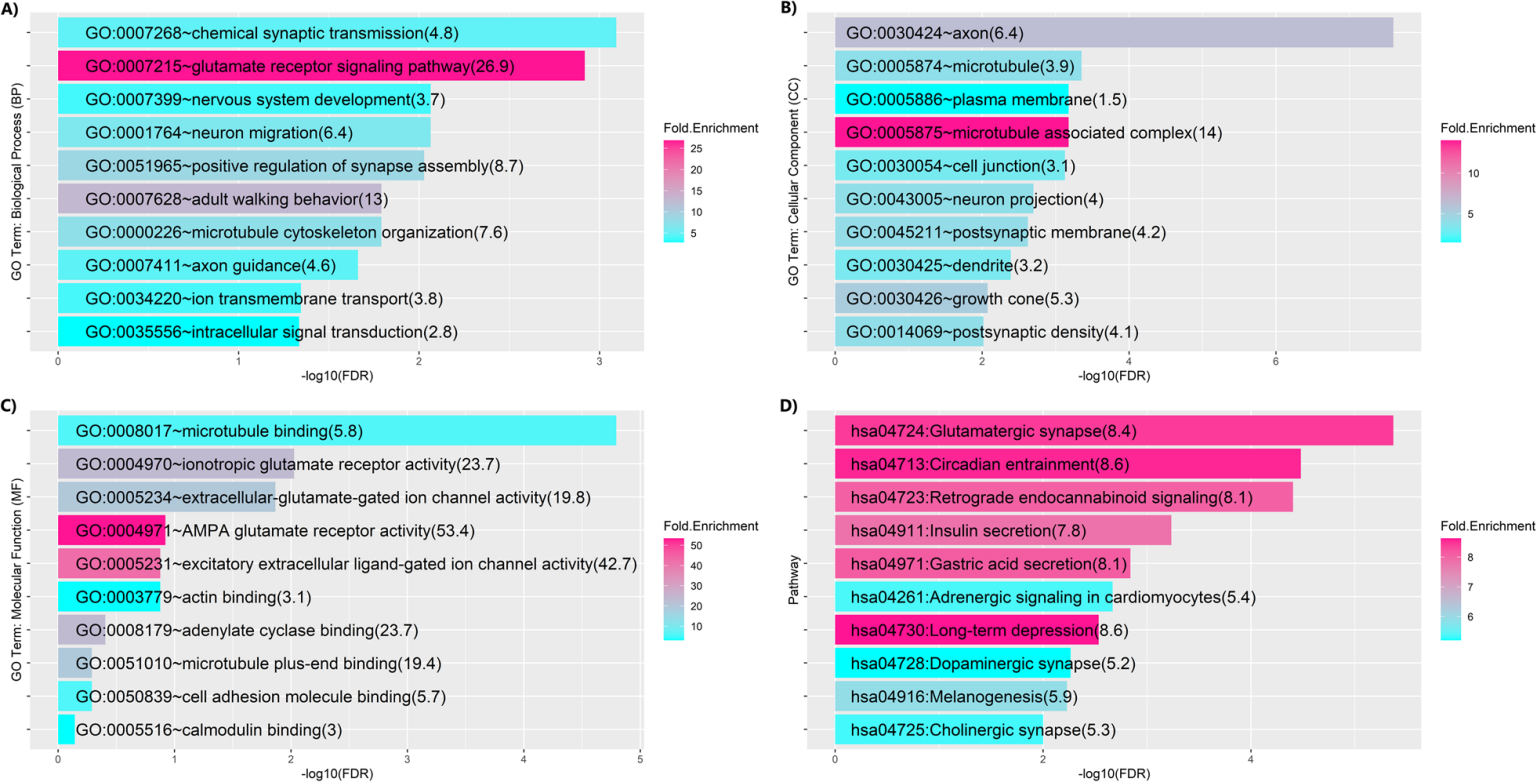
Gene Ontology results for genes significantly co-expressed with *OBI1-AS1.* The color intensity of each bar indicated the fold enrichment score in that GO term. In addition, the exact score is shown in parentheses. FDR values were calculated based on the Benjamini–Hochberg adjustment method. Total number of genes was 293

Gene Ontology results for *OBI1-AS1* are presented in Fig. 5. The top significant GO terms in biological process (BP) (Fig. 5A) were pertinent to synaptic formation and signal transduction. This was compatible with other GO terms in molecular function (MF) and cellular component (CC). GO: CCs (Fig. 5B) showed that most of the genes were located in the plasma membrane of neurons especially in axon, neural projections, dendrites, synapses, and postsynaptic density. Microtubules were another cell compartment where significant number of genes were located. Interestingly, the most significant molecular function GO terms were related to the microtubule binding domains which have an important role in formation of axon and postsynaptic density complexes (Fig. 5C). Other GO: MF terms were linked to glutamate and ionotropic glutamate receptors or ion trafficking across the neural membrane, which indicate that most of the *OBI1-AS1* co-expressed proteins have a binding domain for glutamate and ions like calcium. When we conducted pathway enrichment analysis, hsa04724: Glutamate synapse was found to be the most significant pathway (Fig. 5D). That’s an interesting result because the greatest fold enrichment in BP and MF was also attributed to glutamate receptors. This signaling pathway has a crucial role in response to glutamate in postsynaptic neurons at the postsynaptic density. Some other signaling pathways which were significantly enriched were related to circadian rhythm, secretion and synaptic communication (find the complete list of GO terms and pathways in Additional file 9).

In summary, most GO terms and pathways were attributed to signal transduction at synapses (especially glutamate) and formation of synapses and axons. These are consistent with well-known roles for astrocytes in synapse formation and maintenance of glutamate toxic effect and axon guidance. We emphasize that these findings are based on bioinformatics analysis and functional studies are necessary to make a definite statement in this regard.

## Discussion

In this study, results of bulk RNA-Seq analysis revealed that *OBI1-AS1* has higher expression in LGG samples compared with GBM specimens and this was confirmed by qRT-PCR. In addition, patients with low expression levels of *OBI1-AS1* demonstrated poor prognosis. Also, we showed that this gene was downregulated after glioma recurrence. These findings suggest association of *OBI-AS1* with glioma progression. Furthermore, single-cell RNA sequencing revealed that expression of *OBI1-AS1* is confined to astrocytes. That is an attention-worthy finding which introduces *OBI1-AS1* as a candidate marker for distinction of astrocytes from other brain cell types during cell annotation in the single-cell analysis process.

Astrocytes are known as principal glial cells involved in maintenance of brain homeostasis. Synaptic support, neural protection from glutamate cytotoxic effects, and contribution to axon guidance and synaptogenesis are among some of the most pivotal astrocyte functions. Since very little is known about *OBI1-AS1,* precise elucidation of its functions in astrocytes remains elusive. Hence, GO term analysis of *OBI1-AS1* co-expressed genes is currently the only tool that can be used to speculate on plausible functions this gene fulfills. Our pathway enrichment analysis revealed that *OBI1-AS1* has a potential role in glutamate receptor signaling and synaptic responses, which is compatible with astrocyte functions. Synaptic long-term depression is another attractive pathway which was enriched in our analysis. Some studies in recent years have attributed pathophysiology of depression and mood disorders to astrocytes [38–40]. As previously mentioned, our pathway analysis findings for *OBI1-AS1* were pertinent to astrocyte functions, which was not an unusual finding given the scRNAseq results. These findings can possibly be attributed to the fact that this gene is expressed solely in astrocytes, justifying the existing overlap between GO findings and astrocyte functions. Thus, at this point, no assertion can be made regarding whether there is a causative relationship between GO terms and *OBI1-AS1* function, and further functional studies are required. However, pathway enrichment of *OBI1-AS1* co-expressed genes can currently be used to propose possible signaling pathways *OBI1-AS1* is implicated in, which can be helpful for future functional studies.

### Why is OBI1-AS1 downregulated in GBM and what is the molecular mechanism behind its regulation in glioma?

One possible explanation for the major difference in expression of *OBI1-AS1* in LGG versus GBM is probably the small number of astrocytes found in GBM samples, which is consistent with snRNA-seq results. If this is true, expression of other astrocyte markers in GBM should also be decreased. In Additional file 14: Figure S5, we have presented expression of astrocyte markers in both groups. As demonstrated, expression of these markers (except *OBI1-AS1*) did not show any significant difference between LGG and GBM. It does not seem that the difference in expression of *OBI1-AS1* between the two groups is due to the difference in the number of astrocytes because the expression of other astrocytic markers does not differ much between the two groups. Further exploration of *OBI1-AS1* expression in LGG at single-cell level can clarify this. Unfortunately, we were not able to find such data. Perhaps future studies will help to resolve this issue.

It is known that GBM is comprised of a greater proportion of cancer stem cells than low-grade gliomas. Thus, GBM tumors display greater cellular heterogeneity, higher invasive properties, and, consequently, a more aggressive phenotype [41, 42]. On the other hand, majority of cells in low-grade tumors differentiate into an endpoint glial lineage like astrocytes, oligodendrocytes or a mixture of the two. Since we know that *OBI1-AS1* is exclusively expressed in astrocytes, it is reasonable to assume that *OBI1-AS1* exhibits higher expression in LGG than in GBM, as was the case in our results (Fig. 1A–C). One possible scenario is that upregulation of *OBI1-AS1* in glial stem cells results in differentiation of these cells to astrocytes.

As mentioned previously, *OBI1-AS1* and its surrounding region are significantly hypermethylated in LGG compared to GBM. This is somewhat unexpected, as it is generally believed that methylation usually suppresses gene expression. Since the genome is globally hypermethylated in LGG patients, increased expression of *OBI1-AS1* and hypermethylation at this locus in LGG could be a coincidence. On the other hand, one might entertain the idea that there might be a causative relationship between methylation level at these CpG sites and expression of *OBI1-AS1*. This possibility is reinforced when we know that these CpGs overlap with CTCF binding sites in this region. Flavahan and Drier demonstrated that methylation of CTCF binding sites in IDH1^mut^ samples was the main reason for the difference in expression profile of IDH1^mut^ versus IDH1^wt^ in samples [43]. They showed that CTCF regulate enhancer-promoter contact by managing chromatin interactions. These sites are highly sensitive to methylation and hypermethylation and can disrupt CTCF-DNA interaction and TAD structure [44–46]. If this is the case, expression of *OBI1-AS1* should increase in *IDH1*^*mut*^ compared to *IDH1*^*wt*^ specimens. Moreover, a positive correlation between methylation of CpGs on CTCF binding sites and *OBI1-AS1* expression will be expected. As we showed in Fig. 4C, B, *OBI1-AS1* has higher expression level in IDH1^mut^ samples, and methylation of CTCF binding sites had a positive correlation with *OBI1-AS1* expression. All findings strongly suggested that CTCF served an important role in modulation of *OBI1-AS1* expression. Presence of a strong TAD boundary at midpoint of *OBI1-AS1* in GBM samples, where CTCF bind, supports this hypothesis. This boundary can prevent transcription of this gene by preventing the effect of potential downstream enhancers of *OBI1-AS1* with its promotor or creating a physical barrier to RNA polymerase movement along the gene. Based on available evidence, this is the best explanation that can be provided presently. In the current study, we demonstrated that TAD boundary formation correlates with the expression pattern of this lncRNA. Although these correlations were strong, specific functional studies are required to prove this regulatory mechanism in the future. This study, for the first time, is providing preliminary evidence for significance of this non-coding RNA, but accurate understanding of the regulatory mechanisms affecting this gene require further research.

## Conclusion

For the first time in this study, we presented data to implicate of *OBI1-AS1* in astrocytes and glioma tumors. This gene shows extremely tissue-specific expression pattern which is of great importance. This emphasizes the role of *OBI1-AS1* in astrocytes, but the exact role of this gene in these cells remains an open question that makes this gene an attractive target for future studies on glial cells.

## Supplementary Information

**Additional file 1.** Samples information.

**Additional file 2.** Complete list of DELncRNAs (LGG vs GBM).

**Additional file 3.** The result of DEA using all recurrent LGG samples (after recurrence vs before recurrence).

**Additional file 4.** List of markers for each cluster in Fig.3.

**Additional file 5.** The result of methylation analysis (LGG vs GBM).

**Additional file 6.** Result of DEA based on IDH1 mutation status ( IDH1^mut  ^vs IDH1^wt ^).

**Additional file 7.** The result of  ChIP-seq peak enrichment around *OBI1-AS1* promoter.

**Additional file 8.** The result of methylation analysis based on IDH1 mutation status (IDH1^mut ^ vs IDH1^wt^)

**Additional file 9.** Complete list of GO terms.

**Additional file 10: Figure S1.** Hierarchical analysis of 120 brain cell types used in single nuclei analysis. As illustrated, cells with more biological similarity were in the nearest nodes, meaning that upstream processes do not misrepresent valuable biological features. For example, the three types of astrocytes were the nearest neighbors in the dendrogram (rows 8,9, and 10). Expression of *GFAP* and *S100B* confirmed that these cells are astrocytes. As is presented*, OBI1-AS1* is purely expressed in these three cell types.

**Additional file 11: Figure S2.** Astrocyte Marker gene expression projected on t-SNE.

**Additional file 12: Figure S3.** t-SNE and feature plot of *OBI-AS1* for 4 GBM samples.

**Additional file 13: Figure S4.** Chromatin accessibility (ATAC-seq) and histone modifications (H3K4me3 and H3K27ac) around *OBI1-AS1* promoter. Orange transparent rectangle indicate ± 10 kb around *OBI1-AS1*’s TSS. The pink and blue boxes show the TADs for the Astrocyte and GBM samples, respectively.

**Additional file 14: Figure S5.** The expression of astrocyte markers in GBM and LGG. If the number of astrocytes in GBM is very different from LGG, the expression of other astrocyte markers should also show a significant difference between the two groups.

## Data Availability

The datasets used in the current study are publicly available, and the accession numbers are mentioned in Additional file 1. The codes will be provided at reasonable request directed to the corresponding author.
