# Supplementary figures and images for: Data mining of bulk and single-cell RNA sequencing introduces OBI1-AS1 as an astrocyte marker with possible role in glioma recurrence and progression
Source: Clin Epigenetics. 2022 Mar 8;14:35. doi: 10.1186/s13148-022-01260-4 (PMC8905821; doi:10.1186/s13148-022-01260-4)

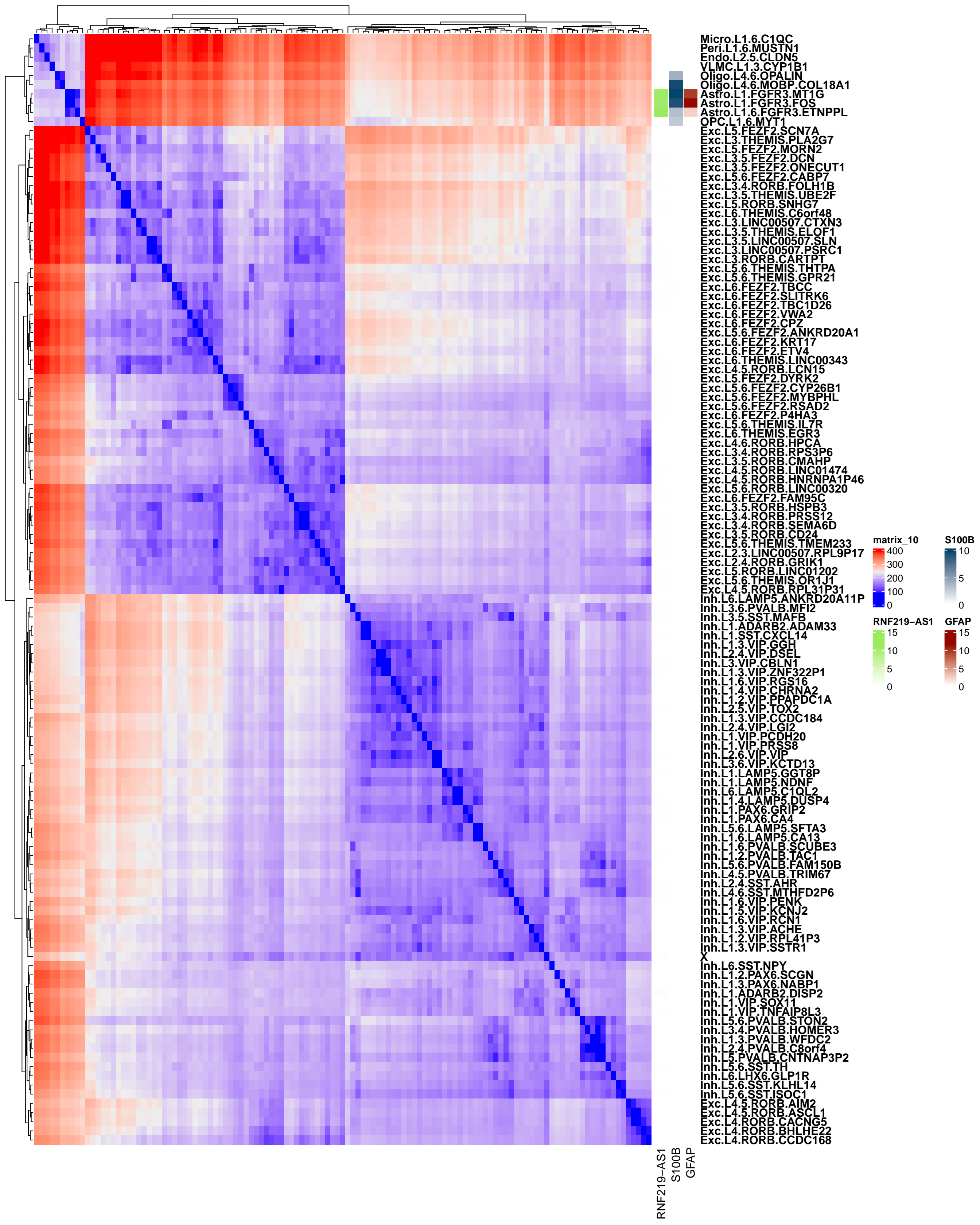

Supplement: Supplementary file 10 — Additional file 10: Figure S1. Hierarchical analysis of 120 brain cell types used in single nuclei analysis. As illustrated, cells with more biological similarity were in the nearest nodes, meaning that upstream processes do not misrepresent valuable biological features. For example, the three types of astrocytes were the nearest neighbors in the dendrogram (rows 8,9, and 10). Expression of GFAP and S100B confirmed that these cells are astrocytes. As is presented, OBI1-AS1 is purely expressed in these three cell types. [file 13148_2022_1260_MOESM10_ESM.pdf]

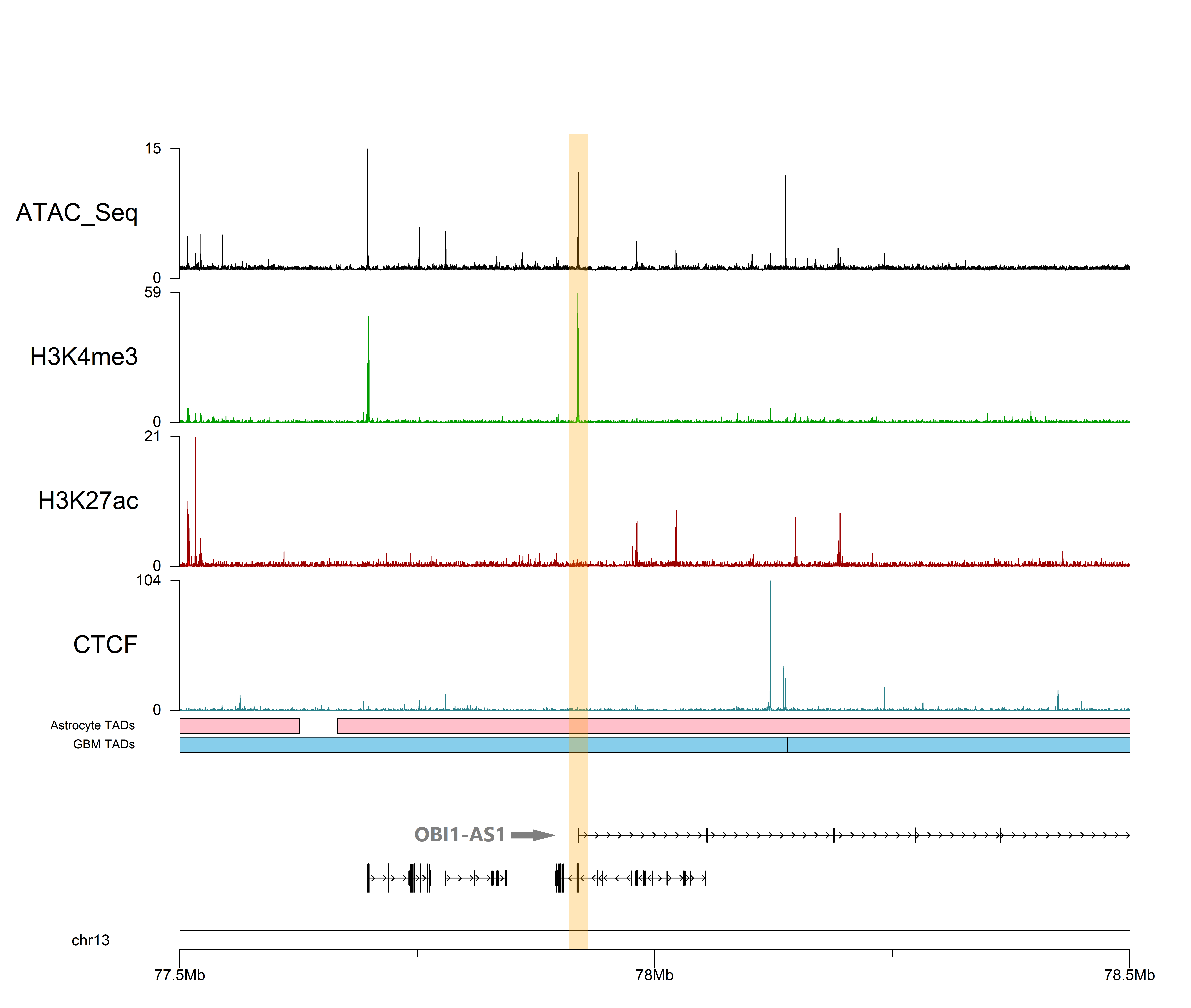

Supplement: Supplementary file 13 — Additional file 13: Figure S4. Chromatin accessibility (ATAC-seq) and histone modifications (H3K4me3 and H3K27ac) around OBI1-AS1 promoter. Orange transparent rectangle indicate ± 10 kb around OBI1-AS1’s TSS. The pink and blue boxes show the TADs for the Astrocyte and GBM samples, respectively. [file 13148_2022_1260_MOESM13_ESM.tif]
